# Adult Disorganized Attachment scale (ADA): Persian adaptation, validity, and reliability study

**DOI:** 10.3389/fpsyg.2025.1471538

**Published:** 2025-05-22

**Authors:** Mitra Pahlavan, Youkhabeh Mohammadian, Nasrin Jaberghaderi, Behzad Mahaki

**Affiliations:** 1 Student Research Committee, Kermanshah University of Medical Sciences, Kermanshah, Iran; 2 Kermanshah University of Medical Sciences, Kermanshah, Iran

**Keywords:** Persian adaptation, validity and reliability study adaptation, adult romantic relationships, disorganized attachment, reliability

## Abstract

**Objective:**

The aim of the current study was to adapt the Adult Disorganized Attachment Scale (ADA) to Persian and to examine its psychometric properties.

**Method:**

The study was conducted with 202 participants, including 124 female (61.4%), who were students at Kermanshah University of Medical Sciences, aged 17–36 years. The Experience in Close Relationships measure was used to assess convergent validity, while the Sadism Scale and Shutdown Dissociation Scale were used to evaluate criterion validity. The reliability using Cronbach’s alpha coefficient, convergent validity, and construct validity using confirmatory factor analysis were examined. The collected data were analyzed in SPSS v.25 and LISREL 8.8 applications.

**Results:**

The scale demonstrated strong reliability (*α* = 0.82) and good convergent validity, with positive correlations to Shutdown Dissociation, Anxious and Avoidant Attachment, and Sadism (*p* < 0.05). Confirmatory Factor Analysis supported its structural validity with excellent fit indexes (χ^2^/df = 1.44, RMSEA = 0.047, CFI = 0.98, GFI = 0.96).

**Conclusion:**

This Persian version of the ADA can be considered a valid and reliable scale, which is a short and practical tool that can be used in clinical and research settings in Iran.

## Introduction

Attachment theory, originally formulated by Bowlby (1969, 1973, 1980), primarily focuses on the relationship between an infant and their caregiver (Belsky, 2002).

Through early interactions, the child forms expectations about the world and themselves. These expectations develop into cognitive models of self and others. Bowlby (1988) referred to these cognitive models as internal working models (IWMs), which serve as the foundation for future relationships (Hoffman, 2006; Zhang et al., 2025; Thompson et al., 2021).

Although these models can adapt and change over time, they generally become more fixed as individuals develop (Belsky, 2002). Therefore, the theory offers a comprehensive framework for understanding the development of personality and the capacity for close relationships in adulthood.

Ainsworth (1982) identified three attachment styles in infants: secure, avoidant, and resistant-ambivalent. Main and Solomon (1990) later recognized a fourth group, disorganized, for children displaying inconsistent, bizarre behaviors, such as freezing or acting out, without a clear behavioral pattern (Hoffman, 2006; Main and Solomon, 1986). They also demonstrated that these infants develop multiple, fragmented internal working models (IWMs) of themselves and their primary caregivers (Main and Solomon, 1986; Liotti, 2006). Bowlby (1973) was one of the first to suggest a link between attachment and dissociative psychopathology and noted that emotionally neglected children develop withdrawal as a defense. When exposed to maltreatment, they often rely on dissociation to cope with trauma (Bowlby, 1980; Blizard, 2001). Even when a caregiver is abusive, the child must stay attached (Bowlby, 1969/1982), leading to complete dependence on the same person who causes harm (Fairbairn, 1952; Main, 1981). This conflict can result in a disorganized attachment style (Main, 1981; Main and Solomon, 1986; Main and Solomon, 1990) and the formation of conflicting views of both the self and the caregiver (Liotti, 1992; Liotti, 1999a; Main, 1991). Dissociation acts as a defense, allowing the child to separate nurturing experiences from abuse. This helps preserve a positive view of the caretaker while developing a separate, powerful, but detached sense of self (Blizard, 1997; Blizard, 2001). Ferenczi suggested that any shock or fear involves some level of personality splitting. He observed that when faced with sudden distress, a weak or undeveloped personality does not defend itself but instead identifies with and internalizes the aggressor (Ferenczi, 1988). This process explains how complex trauma and dissociation can fragment the self, leading to the development of sadistic and masochistic self-states.

attachment strategies influence emotion regulation and relational dynamics. Shaver and Mikulincer (2002) suggest that social psychological methods, such as surveys about romantic relationships, can effectively measure attachment in adults, tapping into emotion-regulation strategies like deactivating, activating, and hyperactivating. Secure individuals handle stress well, express emotions openly, and resolve conflicts constructively. Anxious individuals tend to focus on their distress and use coping strategies that worsen their emotions. Avoidant individuals distance themselves from distressing feelings and avoid confronting painful memories. These behaviors reflect the attachment strategies of hyperactivation and deactivation (Belsky, 2002). Individuals with disorganized attachment often employ a combination of these strategies, alternating between hyperactivation and deactivation. For instance, those who use hyperactivation may experience intense, short-lived relationships followed by sudden breakdowns, often blaming external factors for these failures. Meanwhile, individuals who use deactivation strategies may lead more isolated lives, avoiding close connections. This mix of strategies leads to unstable and conflicting emotional regulation (Bateman, 2022; Bateman and Fonagy, 2019).

Given the critical role of attachment in psychological well-being, researchers have developed various instruments to assess adult attachment styles.

The Adult Attachment Interview (AAI) is the gold standard for assessing adult attachment, offering a detailed evaluation of past and present experiences. However, its administration requires extensive training, certification, and significant time, making self-report measures a more practical alternative (Pollak et al., 2008; Meyer and Pilkonis, 2001; Meier and Bureau, 2018).

To address this limitation, researchers have developed self-report measures specifically designed to assess disorganized attachment.

Psychologists frequently use continuous and dimensional self-report tools such as the Experiences in Close Relationships (ECR) questionnaire (Brennan et al., 1998) and its revised version (Fraley et al., 2000).

These instruments commonly assess attachment in adult romantic relationships using two primary dimensions: anxiety and avoidance. Clinical studies occasionally investigate attachment across three dimensions: secure, anxious, and avoidant.

Specific scales developed to measure disorganized attachment include the Adult Disorganized Attachment (ADA) scale (Paetzold et al., 2015), the Disorganized Response Scale (DRS) (Briere et al., 2018), and the Childhood Disorganization and Role Reversal Scale (CDRR) (Meier and Bureau, 2018; Pollard et al., 2023).

The ADA scale assesses attitudes and responses to current close relationships, specifically addressing the construct of disorganized attachment in adults by focusing on fear, confusion about relationships, and distrust of close others. Meanwhile, the DRS aims to measure self-reported disorganized verbalizations, thoughts, and behaviors specifically related to discussing childhood experiences (Briere et al., 2018).

CDRR is developed to assess disorganized attachment in young adults by measuring their current perceptions of childhood disorganized and controlling attachment (Meier and Bureau, 2018; Pollard et al., 2023; Molisa, 2015).

Some researchers (Mikulincer and Shaver, 2007; Simpson and Rholes, 2002) have argued that disorganization may be a form of fearful avoidance, based on the Relationship Questionnaire (RQ). Fearful avoidants are seen as high in both anxiety and avoidance, leading to conflicting behaviors. However, disorganization is not simply a combination of organized strategies but coexists with them. Most social psychologists use dimensional measures like the ECR and do not examine how high levels of both anxiety and avoidance might interact. Based on the developmental literature, disorganization should be considered as a distinct construct that requires further investigation. The Adult Disorganized Attachment (ADA) scale, developed by Paetzold et al. (2015), was created to specifically assess this distinct attachment pattern, distinguishing it from other forms of attachment insecurity (Paetzold et al., 2015).

In this study we explore the construct of adult disorganized attachment, how it can be evaluated, and whether the related variables are similar to those seen in childhood and adolescence, which have not yet been explored in Iran due to the lack of an appropriate measurement tool. Therefore, there is a need for a suitable instrument to assess adult disorganized attachment in our country. This study aims to develop a Persian adaptation of the ADA (Adult Disorganized Attachment) scale and conduct a validity and reliability study.

## Method

### Sample

This study employed a convenience sampling method, selecting participants from Kermanshah University of Medical Sciences during the 2023–2024 academic year. The sample consisted of 124 females (61.4%) and 202 participants in total. Tinsley and Tinsley (1987) suggest having 5 to 10 participants per item for psychometric studies (Kyriazos, 2018), meaning 45–90 participants are needed for a 9-item measure. With 202 participants, the study meets this requirement. Additionally, Kline (2016) states that a minimum of *N* = 100 participants is necessary for Confirmatory Factor Analysis (CFA) (Kyriazos, 2018), confirming that the sample size of 202 is adequate.

Participants were eligible for inclusion if they were enrolled students at Kermanshah University of Medical Sciences during the study period and had experienced at least one close romantic relationship, and no exclusion criteria were applied.

### Procedure

The Ethics Committee of Kermanshah University of Medical Sciences approved this study on December 13, 2023 (IR.KUMS.MED.REC.1402.246). All participants provided written informed consent after being informed about the study’s objectives and assured of data confidentiality.

The original ADA scale was translated into Persian by three clinical psychologists fluent in both English and Persian. Their recommendations were incorporated to finalize the Persian version. A clinical psychologist with advanced English proficiency then back-translated the scale into English. A pilot test with 14 individuals confirmed the clarity and comprehensibility of the Persian version.

Participants were recruited through direct in-person invitations on campus. Those who agreed completed questionnaires and an interview at the university. No incentives were provided for participation.

### Statistic analysis

Descriptive statistics, including mean and standard deviation (for quantitative variables) and frequency and its percentage (for qualitative variables), were used. Internal consistency reliability was assessed using Cronbach’s alpha. Confirmatory factor analysis was applied to examine validity.

## Measures

### Adult Disorganized Attachment scale (ADA)

The Adult Disorganized Attachment Scale (ADA), was originally developed by Paetzold et al. (2015) assesses disorganized attachment in adults. This self-report measure includes 9 items, each rated on a 7-point Likert scale (1 = strongly disagree, 7 = strongly agree), with total scores ranging from 9 to 63. A single-factor structure accounted for 58.76% of the total variance. The scale demonstrated high internal consistency (Cronbach’s *α* = 0.91) (Paetzold et al., 2015).

### The Shutdown Dissociation Scale (Shut-D)

The Shut-D is a brief structured interview designed to assess vulnerability to dissociation as a consequence of exposure to traumatic stressors, based on the defensive cascade model. This scale includes 13 questions on a 4-point Likert scale (Never = 0, Rarely = 1, Sometimes = 2, Often = 3). Factor analysis provided evidence for unidimensionality; the first factor accounted for 43.4% of the variance (eigenvalue 5.65) and the second factor for 8.2% of the variance (eigenvalue 1.07). The questionnaire demonstrated excellent internal reliability (Cronbach’s alpha = 0.89) and high test–retest reliability (r = 0.93). Convergent validity with the Dissociative Experiences Scale (DES) was significant (r = 0.86). The Shut-D score reliably distinguished patients exposed to trauma from healthy control groups and differentiated between diagnostic groups associated with varying levels of trauma exposure. Exposure to traumatic events increases the diversity and frequency of dissociation and is associated with higher severity of PTSD symptoms and depression levels (Schalinski et al., 2015).

### Experiences in Close Relationships scale (ECR_R)

The Revised Experiences in Close Relationships was developed by Fraley et al. (2000) includes 18 items related to attachment anxiety and 18 items related to attachment avoidance. Participants rated each statement on a 7-point scale from 1 (strongly disagree) to 7 (strongly agree) based on how much they agreed. The ECR-R demonstrates high reliability, with Cronbach’s alpha coefficients for the Anxiety and Avoidance scales typically above 0.85 (Fraley et al., 2000).

## Sadism

The Sadism scale, originally developed by Morten Moshagen, Benjamin E. Hilbig, and Ingo Zettler as part of the Dark Core of Personality framework, was validated in its Persian version with a sample of 541 adults from Tehran. It demonstrated excellent reliability (Cronbach’s alpha = 0.91) and strong construct validity through exploratory and confirmatory factor analyses. The sadism subscale uniquely contributes to the overall dark core beyond general dark traits, highlighting its distinctiveness and relevance. This makes the scale a reliable and valid measure for assessing sadism within the context of dark personality traits (Moshagen et al., 2018; Ebrahimi Ghavam, 2020).

## Result

Data from 202 subjects (61.4% female, 38.6% male; mean age = 22.91 years, SD = 3.77, range 17–36) were used for the analysis. All participants were students of Kermanshah University of Medical Sciences, An independent t-test showed no significant difference in disorganized attachment between women (M = 27.24, SD = 10.26) and men (M = 27.23, SD = 11.05), *p* > 0.05. Levene’s test confirmed homogeneity of variances (*p* = 0.05).

### Confirmatory factor analysis (CFA)

Confirmatory Factor Analysis: The fit of the single-factor structure of the Disorganized Attachment Scale was evaluated using the fit indexes: CFI, NFI, NNFI, GFI, AGFI, IFI, RMSEA, and SRMR. Typically, a Chi-square/df ratio less than 3 indicates a good model fit. However, this index is highly influenced by sample size, and values higher than 3 can also indicate a good fit depending on the sample size.

Generally, an RMSEA less than 0.10, an SRMR less than 0.08, and fit indices CFI, GFI, AGFI, IFI, RFI, NFI, and NNFI above 0.90 (values between 0.80 and 0.90 indicate acceptable and marginal fit) and an AGFI above 0.85 indicate acceptable fit for the structural equation model. The fit results of the single-factor model are presented in Table 1.

**Table 1:** Fit indexes for the single-factor structure of the disorganized attachment scale.

Fit indexes	X^2^	df	X^2^/df	SRMR	GFI	RFI	IFI	CFI	AGFI	NNFI	NFI	RMSEA
One-factor	39/13	27	1.44	0.04	0.96	0.94	0/98	0.98	0.93	0.98	0.96	0.047

RMSEA = Root mean square error of approximation, CFI = Comparative fit index, NFI = Normed fit index, NNFI = Non-normed fit index, AGFI = Adjusted goodness of fit index, RFI = Relative fit index, GFI = Goodness of fit index, SRMR = Standardized root mean square residual, IFI = Incremental fit index.

**Figure 1:**
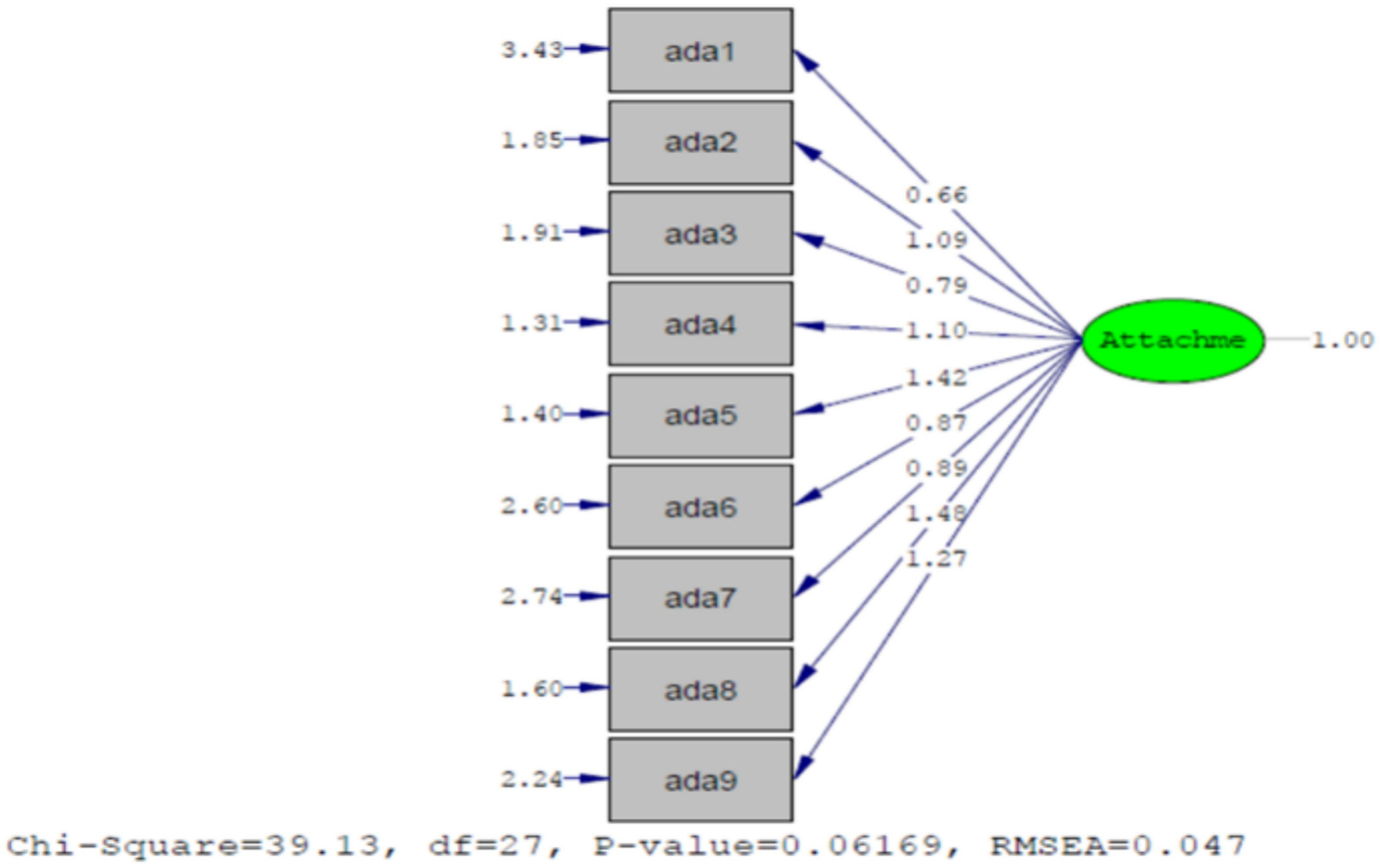
Single-factor model of the disorganized attachment scale. Numbers next to arrows represent factor loadings. Values on the left side of each item indicate error variances.

As shown in the in Figure 1 and Table 2, the single-factor structure of the Disorganized Attachment Scale demonstrates excellent fit.

**Table 2:** Adult disorganized attachment scale factor structure.

Scale items	Mean	SD	Factor loadings	Corrected item-total correlation
1. Fear is a common feeling in close relationships.	4.20	1.96	0.66	0.48**
2. I believe that romantic partners often try to take advantage of each other.	2.89	1.74	1.09	0.66**
3. I never know who I am with romantic partners.	2.25	1.58	0.79	0.58**
4. I find romantic partners to be rather scary.	2.35	1.58	1.10	0.71**
5. It is dangerous to trust romantic partners.	2.76	1.84	1.42	0.74**
6. It is normal to have traumatic experiences with the people you feel close to.	3.58	1.83	0.87	0.57**
7. Strangers are not as scary as romantic partners.	2.99	1.87	0.89	0.57**
8. I could never view romantic partners as totally trustworthy.	3.02	1.94	1.48	0.76**
9. Compared to most people, I feel generally confused about romantic relationships.	3.16	1.96	1.27	0.69**

**Correlation is significant at 0.01 level. *Correlation is significant at 0.05 level. *p < 0.05, **p < 0.01.

### Validity

#### Convergent and criterion validity

Table 3 presents the mean and standard deviation for the variables: Shutdown Dissociation, Disorganized Attachment Style, Anxious Attachment Style, Avoidant Attachment Style, and Sadism. As shown in Table 3, the Disorganized Attachment Scale has a significant positive correlation (*p* < 0.05) with Shutdown Dissociation, Anxious Attachment Style, Avoidant Attachment Style and Sadism, demonstrating good convergent validity for the scale.

**Table 3:** Convergent and criterion validity of the disorganized attachment scale.

Variables	Mean (SD)	1	2	3	4	5
1-Disorganized attachment	27.23 (10.54)	1	0.15*	0.46**	0.47**	0.38**
2-Shutdown dissociation (criterion validity)	7.60 (5.46)		1	0.20*	0.12**	0.28**
3-Anxious attachment (convergent validity)	3.83 (0.93)			1	0.15*	0.29**
4-Avoidant attachment (convergent validity)	3.46 (0.76)				1	0.20**
5-Sadism (criterion validity)	23.60 (10.25)					1

Correlations represent Pearson correlation coefficients. *p < 0.05, **p < 0.01.

##### Regression analysis

A multiple regression analysis was conducted to examine whether attachment anxiety and avoidance predict disorganized attachment. The results showed that the model was statistically significant, F(2, 199) = 61.95, *p* < 0.001, and accounted for 38.4% of the variance in disorganized attachment scores (R^2^ = 0.384, Adjusted R^2^ = 0.378). Both attachment anxiety (*β* = 0.403, *p* < 0.001) and avoidance (β = 0.412, *p* < 0.001) were significant predictors.

##### Reliability

The Disorganized Attachment Scale showed strong internal consistency, with a Cronbach’s alpha of 0.82. The item-total correlations ranged from 0.48 to 0.76, with a mean of 0.65.

## Discussion

The results of the current study indicated that this Persion version of 9-item ADA is a valid and reliable scale.

The results of this study showed single-factor structure of the Persian ADA had a good fit.

The convergent and criterion validity of the Persian ADA were also investigated in our study. The convergent validity was investigated in relation with the experience in close relationships measure and criterion validity was investigated in relation with The Sadism Scale, and Shutdown Dissociation Scale.

The Disorganized Attachment Scale demonstrated good convergent and criterion validity through significant correlations with several related constructs. There was a modest but significant correlation with Shutdown Dissociation (r = 0.15, *p* < 0.05), supporting theoretical expectations that disorganized attachment involves dissociative symptoms. Notably, the scale showed moderate and highly significant correlations with both Anxious Attachment (r = 0.46, *p* < 0.01) and Avoidant Attachment (r = 0.47, *p* < 0.01), indicating that disorganized attachment encompasses aspects of both anxiety and avoidance in attachment behaviors. Paetzold et al. (2015) found higher correlations (r = 0.52 for Anxious Attachment, r = 0.66 for Avoidant Attachment) and reported that anxiety and avoidance together explained 52% of the variance in disorganized attachment (F(2, 496) = 267.74, *p* < 0.001). Similarly, our regression analysis showed that these predictors accounted for 38.4% of the variance (F(2, 199) = 61.95, *p* < 0.001), reinforcing that disorganization is not merely a combination of anxiety and avoidance but a distinct construct.

Additionally, the positive correlation with Sadism (*r* = 0.38, *p* < 0.01) suggests a link between disorganized attachment and sadism, emphasizing the scale’s importance in understanding attachment patterns and their implications for personality disorders and psychopathology.

The reliability analysis of the scale resulted in a Cronbach’s alpha coefficient of 0.82, indicating strong internal consistency among its items. The corrected item-total correlation coefficients of the scale were also in the expected direction and significant. In our study all items ranged from 0.48 to 0.76, which indicates satisfactory values for the scale. The findings indicated that the scale has an acceptable level of reliability.

The results are in line with the factor structure of the main version of ADA in the study by Paetzold et al. (2015).

Our findings are consistent with previous studies using the ADA, which showed strong internal consistency (*α* = 0.882) and significant correlations with disorganized attachment and fearful attachment styles (Pollard et al., 2020).

Additionally, large positive correlations were observed between disorganized attachment and dissociation, further supporting the ADA’s psychometric reliability (Pollard et al., 2020).

Our results are consistent with previous research on the Turkish version of the ADA scale, which showed good psychometric properties and acceptable internal consistency (Cronbach’s alpha = 0.79). Significant correlations with the ECR anxious and avoidant attachment subdimensions further support the scale’s validity (Erturk and Batigun, 2021).

Our study shows that the fit indices for the single-factor structure of the Disorganized Attachment Scale indicate a strong model fit. The SRMR is 0.04, suggesting a good fit, while other indices (GFI, RFI, IFI, CFI, AGFI, NFI, NNFI) range from 0.93 to 0.98, confirming the model’s quality. The RMSEA is also 0.04, supporting the model’s adequacy. These results confirm that the single-factor model is a good fit for the data, demonstrating its validity and reliability in assessing disorganized attachment.

A bunch of research and theories confirm that disorganized attachment results from complex trauma and is correlated with dissociation symptoms (Liotti, 1999b; Burback et al., 2024; Guérin-Marion et al., 2020).

As we mentioned already, in the literature of psychopathology, the common assessment of attachment styles was included in two dimensions: anxious attachment style and avoidant attachment style. In the previous researches, one study that used the two common dimensions, found that sadism is only related to avoidant attachment style (Nickisch et al., 2020), and another study, mentioned that sadism was related to anxious attachment style, while Machiavellianism was related to disorganized attachment style (Russell and King, 2016). our study found a positive correlation between sadism and disorganized attachment (r = 0.38, *p* < 0.01).

In an interesting study, researchers found that anxious maternal attachment indirectly predicted sexual violence (Russell and King, 2016). We recommend similar research that examines disorganized attachment with the ADA scale.

The ADA is a useful tool for assessing disorganized attachment. The main goal of this study was to investigate the validity and reliability of the Persian ADA among students in Kermanshah, Iran. The results of this study showed that the single-factor structure of the Persian ADA had a good fit.

Our results demonstrated that the Persian version of the ADA is a valid and reliable scale for assessing disorganized attachment in adulthood. Due to the lack of Persian measurement tools for assessing disorganized attachment in adult romantic relationships, no local studies have quantitatively addressed this issue. This has represented a significant gap in the Persian literature. The validation of the Persian adaptation of the ADA, as demonstrated by our study, is a crucial step in filling this gap. This scale will be valuable in future research, particularly in studies examining various psychopathologies such as borderline personality disorder, dissociative symptoms, post-traumatic stress disorder, and sadism.

Understanding disorganized attachment in adulthood, how it develops, and how it appears in adult relationships is essential for developing effective interventions (Jacobvitz and Reisz, 2019).

This study has some limitations that should be considered. First, the sample included only university students, which may limit how well the findings apply to the general population. Future studies should include more diverse participants to improve generalizability.

Second, while this study confirmed the initial reliability and validity of the ADA, it did not assess test–retest reliability. Future research should examine whether the scale produces consistent results over time.

Despite these limitations, this study provides valuable evidence for the ADA’s psychometric strength. The use of comprehensive statistical analysis supports its reliability as a measure of disorganized attachment. Future studies should further test its effectiveness in clinical settings and different cultural contexts.

In conclusion, the Persian version of the ADA is a valid and reliable tool for assessing disorganized attachment in adulthood. Its validation fills a critical gap in the Persian literature and paves the way for future research and clinical applications. By addressing the limitations of this study and incorporating additional reliability and validity measures, future research can further enhance our understanding of disorganized attachment and its implications for mental health.

## Data Availability

The raw data supporting the conclusions of this article will be made available by the authors, without undue reservation.

## Author’s note

This article includes a part of the master dissertation study conducted by Mitra Pahlavan under the advisory of Dr. Yukhabeh Mohammadian within the scope of Kermanshah University of Medical Sciences, Clinical Psychology Master Program.
