# Correction: Adult Disorganized Attachment scale (ADA): Persian adaptation, validity, and reliability study

**DOI:** 10.3389/fpsyg.2025.1751064

**Published:** 2025-12-15

**Authors:** Mitra Pahlavan, Youkhabeh Mohammadian, Nasrin Jaberghaderi, Behzad Mahaki

**Affiliations:** 1 Student Research Committee, Kermanshah University of Medical Sciences, Kermanshah, Iran; 2 Kermanshah University of Medical Sciences, Kermanshah, Iran

**Keywords:** Persian adaptation, validity and reliability study adaptation, adult romantic relationships, disorganized attachment, reliability

There was a mistake in Table 3 as published. The published table showed wrong correlations: Shutdown Dissociation with Anxious attachment was *r* = 0.15^*^ and Shutdown Dissociation with Avoidant attachment was *r* = 0.20^**^.

The corrected Table 3 appears below.

**Table 3:** Convergent and criterion validity of the disorganized attachment scale.

**Variables**	**Mean (SD)**	**1**	**2**	**3**	**4**	**5**
1-Disorganized attachment	27.23 (10.54)	1	0.15^*^	0.46^**^	0.47^**^	0.38^**^
2-Shutdown dissociation (criterion validity)	7.60 (5.46)		1	$0.20^{**}$	0.12	0.28^**^
3-Anxious attachment (convergent validity)	3.83 (0.93)			1	0.15^*^	0.29^**^
4-Avoidant attachment (convergent validity)	3.46 (0.76)				1	0.20^**^
5-Sadism (criterion validity)	23.60 (10.25)					1

Correlations represent Pearson correlation coefficients. ^*^p < 0.05, ^**^p < 0.01.

The original version of this article has been updated.

